# Common design and data elements on rectal artery embolization for treatment of symptomatic internal hemorrhoidal disease: an interactive systematic review of clinical trials

**DOI:** 10.1186/s42155-024-00458-2

**Published:** 2024-05-11

**Authors:** Samah Morsi, Marisabel Linares Bolsegui, Hassan Kobeissi, Sherief Ghozy, David F. Kallmes, Scott R. Kelley, Kellie L. Mathis, Eric J. Dozois, Conor G. Loftus, Emily C. Bendel, Vincent Vidal, Scott M. Thompson

**Affiliations:** 1 Department of Radiology, Mayo Clinic, Rochester, MN USA; 2 Department of Neurologic Surgery, Mayo Clinic, Rochester, MN USA; 3 Division of Interventional Radiology, Russell H. Morgan Department of Radiology and Radiological Science, Johns Hopkins Hospital, Baltimore, MD USA; 4 Division of Colon and Rectal Surgery, Mayo Clinic, Rochester, MN USA; 5 Department of Medicine, Division of Gastroenterology and Hepatology, Mayo Clinic, Rochester, MN USA; 6 Department of Vascular and Interventional Radiology, Hôpital de La Timone, Marseille, France

**Keywords:** Hemorrhoids, Rectal artery embolization, Study design, Data elements

## Abstract

**Background:**

Internal hemorrhoids (IH) is a common medical condition that can result in morbidity secondary to bleeding and discomfort. Treatment for IH has traditionally consisted of dietary and conservative medical management, focal treatments including banding and sclerotherapy or hemorrhoidectomy. Recently, rectal artery embolization (RAE) has been studied as a potential treatment for bleeding predominant IH. We performed a common design and data element analysis of studies that report on RAE.

**Materials and methods:**

We conducted a qualitative systematic literature review for rectal artery embolization (RAE) for symptomatic hemorrhoidal disease. The screening process involved five online databases (PubMed, Embase, Google Scholar, DOAJ, and Scopus). Additionally, ClinicalTrials.gov was examined for active, unpublished completed studies. The initial search yielded 2000 studies, with 15 studies meeting the inclusion criteria after screening and assessment. The included studies comprised one RCT, one case series, one pilot study and 12 cohort studies.

**Results:**

The population analysis revealed a male predominance across all studies, with varying cohort sizes. The baseline Goligher hemorrhoid grade was utilized in 80% of studies. The majority (73.3%) employed a transfemoral approach, and coils were the primary embolic material in 60% of studies, 26.6% were combination of coils and particles, and 6.6% were particles only. Patient selection criteria highlighted RAE's applicability for high surgical risk patients and those with anemia, chronic hematochezia, or treatment-refractory cases. Exclusion criteria emphasized factors such as previous surgeries, colorectal cancer, rectal prolapse, acute hemorrhoidal complications, and contrast allergy.

Study designs varied, with cohort studies being the most common (12/15; 80%). Procedural details included the use of metallic coils and detachable micro-coils, with a high technical success rate reported in most studies ranging from 72 to 100%. The follow-up ranged from 1 to 18 months. The majority of studies reported no major immediate or post-procedural complications.

**Conclusion:**

While all studies focused on RAE as a treatment for IH, there was a great degree of heterogeneity among included studies, particularly regarding inclusion criteria, exclusion criteria, outcomes measures and timeframe. Future literature should attempt to standardize these design elements to help facilitate secondary analyses and increase understanding of RAE as a treatment option.

**Supplementary Information:**

The online version contains supplementary material available at 10.1186/s42155-024-00458-2.

## Introduction

Hemorrhoids (HD) are the fourth most prevalent gastrointestinal diagnosis in adults in outpatient settings and the most common cause of anal pathology, contributing to approximately 3.3 million visits for ambulatory care in the United States every year. The self-reported annual occurrence of hemorrhoids in the country is around 10 million cases, representing approximately 4.4% of the total population [1]. HD can manifest with various symptoms, such as anal pruritis, pain, swelling, and bleeding [2].

Several risk factors contribute to the development of HD, including straining during bowel movements, prolonged sitting, chronic diarrhea or constipation, obesity, pregnancy, anal intercourse, a low-fiber diet, and heavy lifting. HD significantly impacts patients' quality of life due to physical discomfort, potentially requiring lifestyle adjustments [2, 3]. Additionally, acute severe hemorrhoidal bleeding can result in acute blood loss anemia that may require hospitalization, hemodynamic support and blood transfusion while chronic blood loss anemia may necessitate oral or intravenous iron supplementation ± blood transfusion.

The use of the patient-reported outcome measures (PROMs) to evaluate the effect of HD symptoms on daily life provides a deeper understanding of the disease's burden by collecting direct patient feedback. This approach aids clinicians in better addressing patients' experiences with the condition [2].

Hemorrhoids (HD) are either internal hemorrhoids (IH) or external hemorrhoids (EH). IH and EH differ primarily in their location and the symptoms they produce. IH are situated inside the anus, typically causing painless rectal bleeding and, in more severe cases, prolapse during bowel movements. In contrast, EH can extend up to the dentate line. They can involve both the anal verge and the first half of the anal canal and are often associated with symptoms like pain, itching, swelling, and the potential formation of painful blood clots (thrombosis).. These distinctions in location and symptoms are crucial in the diagnosis and treatment of hemorrhoids, helping healthcare professionals determine the appropriate management strategies for each patient's specific condition [4].

The American Society of Colon and Rectal Surgeons (ASCRS) and the European Society of Coloproctology (ESCP) have established evidence-based clinical practice guidelines for the diagnosis and management of symptomatic hemorrhoidal disease, which were updated in 2024 and 2020, respectively [5, 6]. These guidelines outline a systematic approach to the diagnosis of hemorrhoidal disease, as well as a treatment approach based on hemorrhoidal grades (I–IV) in the Goligher classification system. There are numerous medical (dietary measures, phlebotonics), office-based procedural (rubber band ligation [RBL], infrared photocoagulation, bipolar diathermy, sclerotherapy), and surgical (hemorrhoidectomy, hemorrhoidopexy, transanal hemorrhoidal dearterialization) treatment options for hemorrhoidal disease with well-established safety and efficacy profiles [1]. However, high recurrence rates of bleeding are not uncommon after standard of care therapies which has prompted investigation into new therapeutic approaches.

Histopathologic evaluation has identified that hemorrhoids are vascular structures arising from channels of arteriovenous connective tissues with arterial supply from the superior, middle and/or inferior rectal (hemorrhoidal) arteries and drain into the superior and inferior hemorrhoidal veins [7]. Based on this observation that there are arteriovenous connections within hemorrhoids with an arterial inflow to the corpus cavernosum recti, doppler guided hemorrhoidal artery ligation (HAL) was introduced as a treatment option for IH. Doppler-guided hemorrhoid artery ligation is performed via an anoscope with a Doppler probe that is used to localize hemorrhoidal artery branches for directed transanal suture ligation [8]. Building on the principles of hemorrhoidal dearterialization from doppler guided HAL, trans-arterial catheter-directed rectal artery embolization (RAE; “emborrhoid”) was first described by Vidal et al. in 2014 as a treatment option for internal HD [9, 10]. Over the next decade promising data have emerged on the technique, safety, and efficacy of rectal artery embolization for treatment of bleeding predominant IH [3, 9]. Recent meta-analyses of clinical studies have shown high technical success (98–99%) and short-term clinical efficacy to 1-year (79–82%) with clinically significant improvements in bleeding scores, quality of life and pain scores reported [10, 11]. Importantly, no bowel ischemia, necrosis or anorectal compilations have been reported [10, 11]. However, questions have remained regarding patient selection, the optimal embolization technique and method of outcome response assessment.

Given the novelty of RAE, the common design and data elements in clinical trials or research studies remain in the early stages of development. There is limited information available regarding standardized protocols, study designs, or data elements. Further research and clinical trials are necessary to establish these elements, evaluate the safety and efficacy of RAE, and compare it with other available treatment options for hemorrhoidal disease [1, 9, 10].

The purpose of this study is to collate data elements and outcomes in clinical trials to highlight the heterogeneity in the evidence of RAE for hemorrhoidal disease, aiming to promote standardization in future research to facilitate systematic reviews and meta-analyses.

## Materials and methods

### Literature search

We conducted a qualitative systematic literature review, following to the Preferred Reporting Items for Systematic Reviews and Meta-Analyses (PRISMA) guidelines and using the {Nested} Knowledge (NK) AutoLit living semi-automated systematic review platform (St Paul, Minnesota, USA(R)) for conducting the search [12–14]. The NK AutoLit platform searching function enables exploring an extensive database of records using specific keywords. This creates an orderly framework for conducting literature reviews and meta-analyses, ensuring robust, reliable, and reproducible processes [15–17]. We queried five online databases (PubMed, Embase, Google Scholar, DOAJ, and Scopus) using the search terms ' ((Rectal artery) AND (Embolization OR Emborrhoid OR percutaneous OR transcatheter)) AND (Hemorrhoids OR Hemorrhoidal disease OR haemorrhoidal disease) ' on November 20, 2022. Additionally, we complemented the search of published studies, examining thoroughly ClinicalTrials.gov looking for active, unpublished completed studies, and preliminary findings. The identification process was further enhanced by manually scrutinizing reference lists of eligible studies and pertinent review articles to uncover any studies that may have been overlooked during the database search. As this study is confined to publicly accessible data, Institutional Review Board (IRB) approval was not required.

### Inclusion & exclusion criteria

**Fig. 1:**
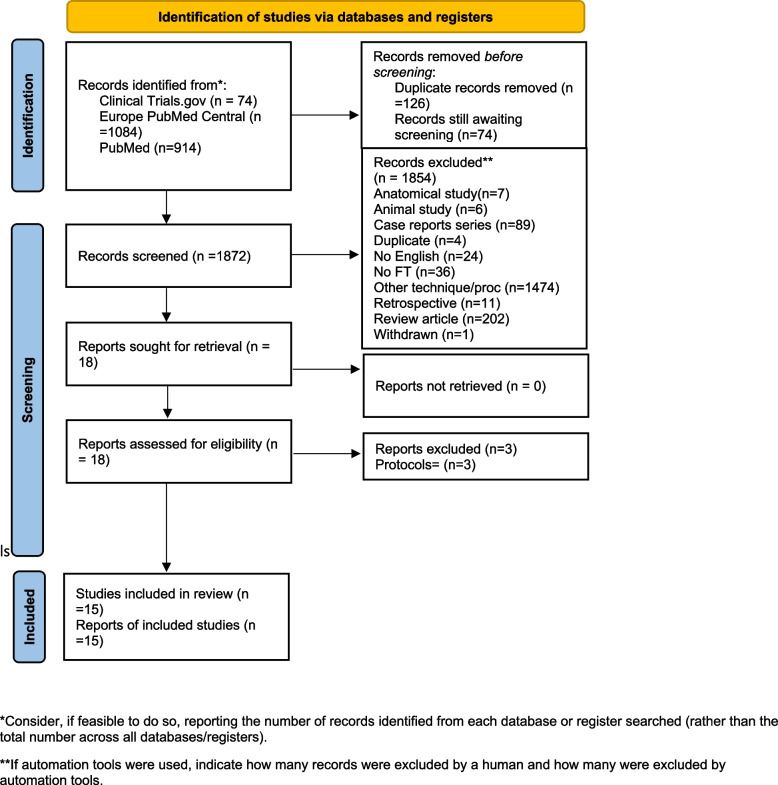
PRISMA flow diagram detailing the literature search process

The screening was performed by two independent reviewers, initially evaluating study titles and abstracts for relevance. Subsequently, full texts or protocols of the preliminarily included articles were assessed for eligibility. Any discrepancies were resolved through discussion with a third senior author to reach consensus, based on the following inclusion criteria: full text in English, randomized clinical trials (RCTs) or prospective cohort studies examining clinical outcomes of patients with symptomatic hemorrhoidal disease undergoing RAE, either as a standalone treatment or in comparison with other interventions (sham procedure, conservative management, hemorrhoidectomy, or rubber band ligation). Excluded records consisted of literature reviews, case reports, and non-human studies, anatomical studies, other surgical techniques for hemorrhoidal disease treatment or lacking study endpoints, and those with republished data involving patients already included in the analysis (Fig. 1).

#### Tagging common data elements

All reported data elements were tagged through the AutoLit tagging feature. We conducted an exhaustive analysis of the included studies to identify predetermined outcomes, along with their corresponding definitions and specific follow-up time points. The NK AutoLit feature streamlined the extraction of qualitative data using tags, allowing the identification and retrieval of critical information during the review. Distinct tags were created for each data element based on a the full text or full protocol, as appropriate, and two authors completed the tagging process, with an independent author reviewing it for quality assurance. Additionally, sub-tags were generated to depict records that were incomparable when encountering unique or non-combinable definitions associated with each data element.

#### Data synthesis and analysis

**Fig. 2:**
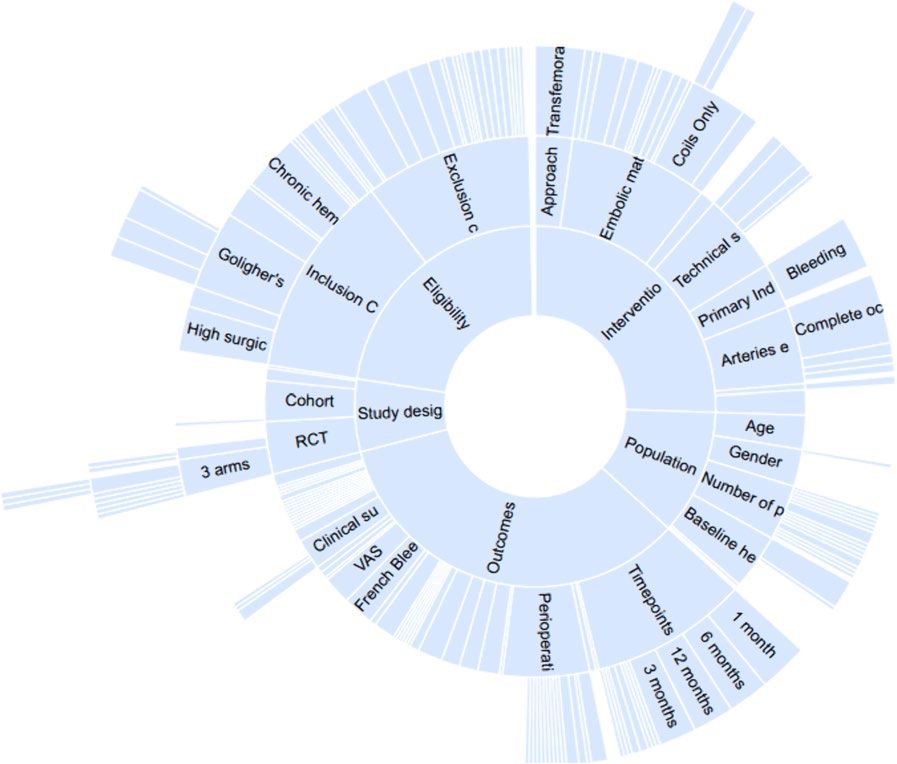
Sunburst diagram of data elements in the Nested Knowledge (NK) nest for this study. The frequency of each data element can be viewed by clicking on it, along with frequently co-occurring tags. In this example,’Cohort study’ is a tag associated with 18 of the studies, and co-occurring tags include Pain (co-occurring in all cohort studies) and Kellgren-Lawrence grade (co-occurring in 17 of the 18 cohort studies). Clicking on each data element outputs a frequency of the tag associated with it, as well as frequently co-occurring tags. See https:// nested-knowledge.com/nest/qualitative/2499 for an interactive version of this figure

After concluding the tagging process, the NK qualitative synthesis feature allowed the visualization of the frequency of study design types and data elements using a sunburst diagram. Each sunburst section represents a tag found across trials, with the occurrence of each tag denoted in the platform based on the number of tagged elements among all the studies. Furthermore, the size of each segment indicates the frequency of that tag based on the number of studies it appears in. Consequently, tags present in multiple studies are represented by larger sections in the diagram, while those found in fewer studies appear as smaller sections. This visualization tool offers an overview of the prevalence of each data element (tag) across the studies reviewed (see Fig. 2; for the interactive version, visit https://nested-knowledge.com/nest/qualitative/4809). Once the data was extracted and analyzed from the NK qualitative synthesis feature, tables and figures were produced using Microsoft presentation software and GraphPad Prism software.

### Risk of bias and quality assessment

While conventional risk-of-bias assessments for randomized controlled trials (RCTs) and quality score evaluations for observational studies are common practices in systematic reviews, recommended by the PRISMA guidelines, the scope of this review required a nuanced approach. Our primary objective does not involve assessing the outcomes of specific interventions, instead, the analysis of methodologies, reporting practices, and identification of common data elements in clinical trials of superior RAE as treatment for hemorrhoidal disease. The intent is to enhance consistency, transparency, and future comparability of trial outcomes among studies.

## Results

### Search results

The initial search yielded 2000 studies, of which 124 were removed as duplicates (Supplementary Table 1). Subsequently, 1872 records were screened for inclusion; 1837 were excluded based on title and abstract, and 35 were assessed for eligibility. Ultimately, 15 studies that fulfilled the inclusion criteria were tagged and incorporated into the final analysis (Fig. 1, Supplementary Table 2). There was one RCT, one case series, one pilot study and 12 cohort studies (Supplementary Fig. 1).

### Overview of included studies

#### Population

In the analysis of the population characteristics across the selected studies, there was a male predominance, as reported in 100% (15/15) of the studies. The size of the study cohorts varied widely, with participant numbers ranging from 12 individuals in 20% (3/15) of the studies (Lezzi R. et al., Tradi F. et al., and Küçükay F., et al.) to a cohort of 80 participants in the HEMbol study (NCT05697562). The Goligher Baseline hemorrhoidal grade was utilized in 80% (12/15) studies.

### Intervention

Among the studies, 73.3% (11/15) employed a transfemoral approach, while details of the intervention method were not provided in 46.6% (7/15) of the investigations. In the embolic category, coils were exclusively utilized in 60% (9/15) of the studies, with two investigations specifying the type and size of the coils (Vidal V. et al.) and (Moussa N. et al.). Furthermore, fibered coils (Falsarella PM. et al.) were employed in 20% (3/15) of the studies, featuring detachable coils. Additionally, Gelfoam and gelatin sponge particles were utilized in 6.6% (1/15) of the studies, while Embosphere was used in 13.3% (2/15) of the investigations (Küçükay F. et al., and Stecca T. et al.).

### Common design and data elements

1. Patient selection criteria

**Fig. 3:**
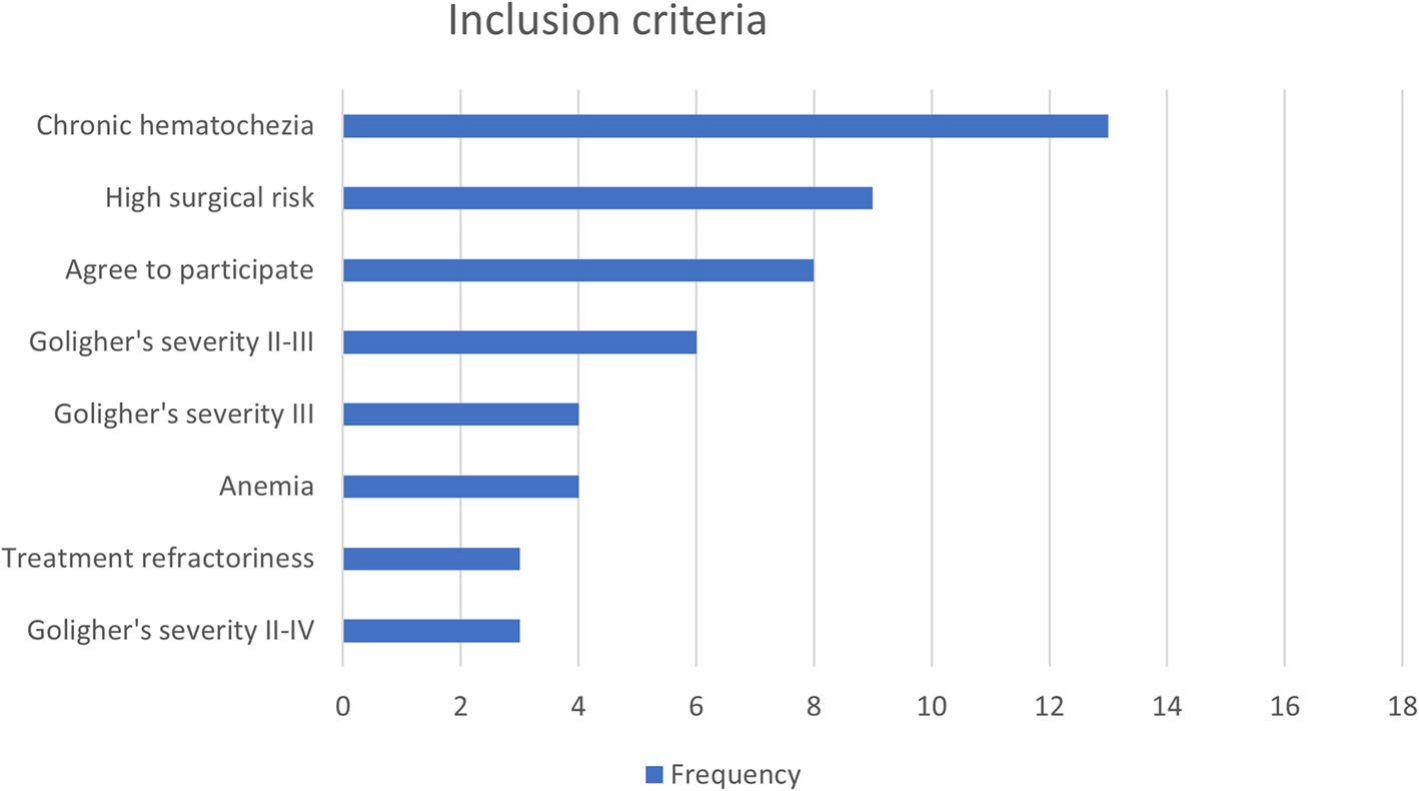
Tag frequencies and eligibility inclusion in the study population

RAE was found to be an intervention for patients at high surgical risk, with 9 studies highlighting its potential in this context. Additionally, 4/ 15 studies (26.6%) emphasized RAE's applicability for individuals with anemia. The classification of hemorrhoidal disease severity according to Goligher's criteria was also explored, with 4 studies focusing on grade III, 3 studies for grades II-IV and 6 studies on II-III grades levels. Moreover, RAE garnered attention as an alternative for cases of chronic hematochezia in 13 studies and exhibited potential in addressing treatment-refractory situations, a theme observed in 3 studies. The presence of the "Anemia" tag in 4 trials highlights a significant consideration in the utilization of RAE for symptomatic hemorrhoidal disease. Lastly, the recognition of "Treatment refractoriness" was recognized in 3 studies (Figs. 2 and 3).

**Fig. 4:**
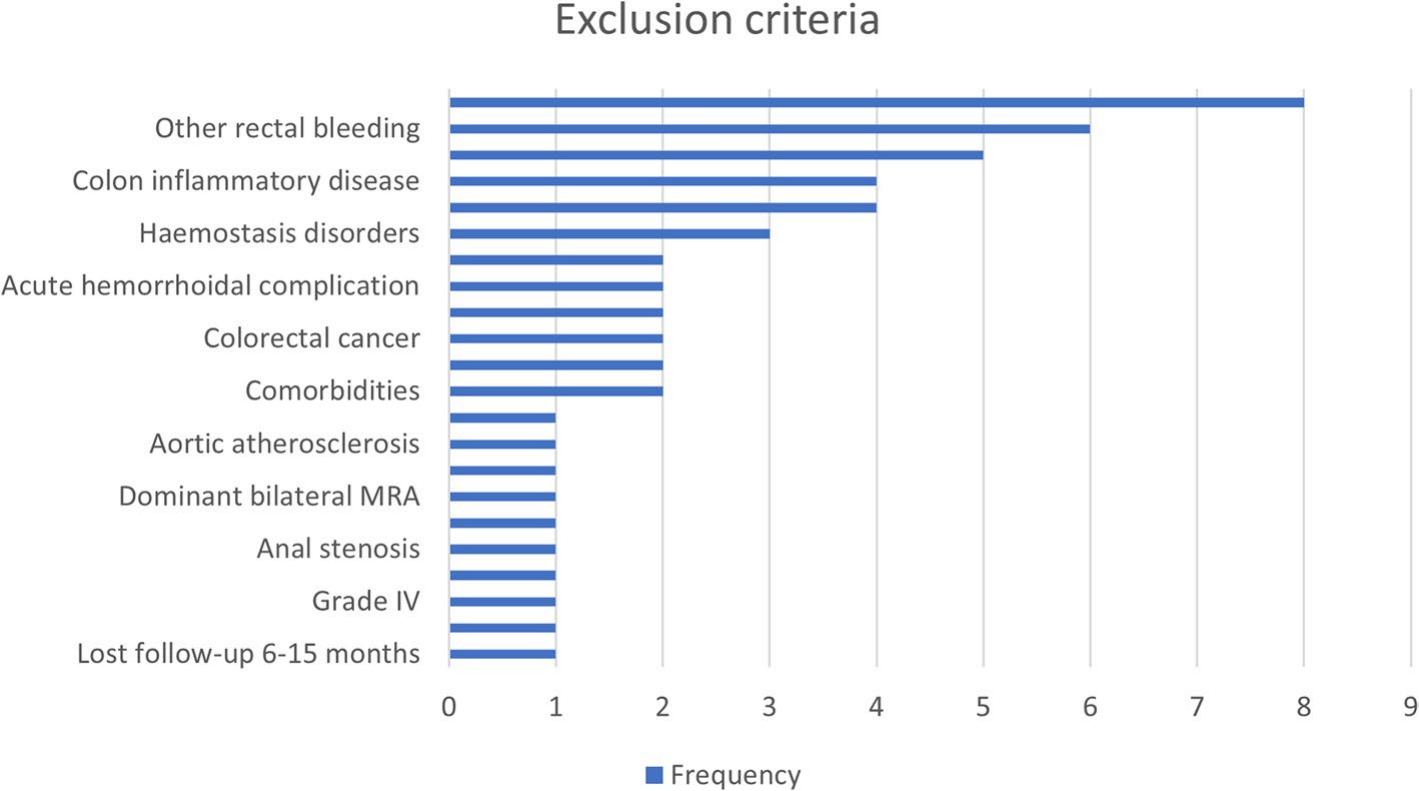
Exclusion criteria and frequency of associated tags in patient eligibility assessment

The examination of "eligibility exclusion criteria" identified important insights that might influence the suitability of patients for RAE. These factors include a history of previous surgeries related to hemorrhoidal disease or superselective rectal Artery embolization (SRAE), with the "Previous SRAE or hemorrhoidal surgery" tag appearing in 4 studies. Furthermore, the presence of conditions such as colorectal cancer, rectal prolapse, acute hemorrhoidal complications, and colorectal inflammatory diseases were associated with exclusion from RAE treatment, as indicated by respective tags appearing 2 or more times. The occurrence of the "Contrast allergy" tag in 8 trials suggests the importance of patient safety considerations during RAE procedures. Collectively, these findings highlight the meticulous patient selection process and the necessity of evaluating various exclusion criteria to ensure the appropriate application of RAE in managing symptomatic hemorrhoidal disease (Fig. 4).

## Heterogeneity in study designs and data elements

The distribution of study design frequencies reveals various approaches employed in investigating RAE for symptomatic hemorrhoidal disease. "RCT" appeared in 1 trial. The frequency of "Protocol" appeared in 3 studies. "Cohort study " was the most common study design, with 12 studies utilizing this design. This diverse array of study designs collectively contributes to a comprehensive understanding of RAE's effectiveness and its potential in managing symptomatic hemorrhoidal disease (Supplementary Fig. 1).

## Procedural details and follow-up duration

The details of the procedure varied significantly among the included studies and are provided in Supplementary Table 3. Metallic coils (5/15 studies, 33.3%) and detachable micro-coils (5/15 studies, 33.3%) were the most common type of embolic material used (Supplementary Fig. 2). The technical success rate was consistently high among studies, with ten (66.6%) studies reporting a technical success rate of 100%. 3 studies (20%) reported more than 90% technical success. However, the technical success rate was not reported in one (6.6%) study (Supplementary Fig. 3). The most common approach was the transfemoral (10/15 studies, 66.6%) (Supplementary Fig. 4). The need for re-intervention was reported among 40% of included studies (6/15 studies, 40%) (Supplementary Fig. 5). The most common follow-up points reported among studies were 6 months (9/15 studies, 60%) and 1 month (9/15 studies, 60%) (Supplementary Fig. 6). Finally, the majority of studies reported no major immediate or post-operative complications (14/15 studies, 93.3%) (Supplementary Fig. 7).

## Treatment outcomes and clinical success rates in internal hemorrhoid management

Various outcome assessments have been utilized to evaluate the efficacy of treatments for hemorrhoids, with clinical success rates serving as a primary measure of effectiveness. Studies such as those conducted by Sun et al. (2018), Puchol et al. (2020), and Küçükay et al. (2021) have consistently reported high clinical success rates ranging from 83.4% to 93%. Clinical success encompasses a range of factors including symptom resolution, improvement in quality of life, and patient satisfaction. Additionally, outcome assessments such as the Goligher's grading system (GPS), French Bleeding Score (FBS), Visual Analog Scale (VAS) for pain, and quality of life scores (QOLS) have been detailed for quantifying treatment outcomes. (Supplementary Table 2).

## Discussion

Our common design and data elements analysis for RAE revealed several important findings. To our knowledge, this is the first common design and data elements analysis for this subject. First, we found that there was a high degree of heterogeneity in the present literature regarding the treatment. The heterogeneity was more prominent in certain categories, while other categories, such as follow-up periods, were more homogeneous.

There is limited published literature regarding study design for RAE. However, common design and data elements analysis has been performed for other treatments, with a more recent focus on the topic from the national institute of health (NIH). Recent common design and data elements analysis for neurointerventional procedures and genicular artery embolization for knee osteoarthritis have identified a similar degree of heterogeneity [18–20].

Because literature regarding RAE is currently in its infancy within the first 10 years since its original description, it is important to identify these gaps in the literature at the present. Future studies should attempt to standardize and homogenize inclusion criteria, exclusion criteria, and follow-up timing and metrics to allow for better interpretation and secondary analyses of the literature. The current degree of heterogeneity represents a constraint towards understanding the efficacy of RAE as an intervention, as interpreting inter-study results is cumbersome and unclear.

RAE represents a unique treatment option due to the opportunity for multidisciplinary involvement in disease management. The intersection of and collaboration between interventional radiology, surgery, gastroenterology, and others is likely to facilitate rapid progress in the treatment of HD. Yet, determining treatment algorithms and referral patterns remains an unmet need in HD. With an increasing amount of literature and treatment options for HD, these gaps in treatment will continue to close. Increasing cohesion among studies will help to hasten the progress on HD. Currently RAE is not included in either the US or European guidelines for treatment for hemorrhoidal disease [12, 13].

The current evidence on rectal artery embolization (RAE) for hemorrhoidal disease is constrained by heterogeneity in patient populations, technical methodologies, outcome measures, and follow-up. Consequently, numerous aspects, including patient selection, embolization technique, and predictive factors for technical and clinical success, have yet to be adequately addressed. These unresolved matters warrant further exploration through prospective clinical trials or registries. As a result, the establishment of common design and data elements for RAE in the context of hemorrhoidal disease remains an ongoing endeavor [1].

## Conclusions

Well-designed multidisciplinary prospective clinical trials or registries are needed to address several issues regarding patient selection, technical factors of embolization, and prognostic factors for technical and clinical success. By conducting such studies, it may be possible to integrate the current evidence and standardize rectal artery embolization (RAE) for hemorrhoidal disease.

### Supplementary Information

Supplementary Material 1.

## Data Availability

The datasets analyzed during the current study are available from the corresponding author on reasonable request.
