# Supplementary material for: Common design and data elements on rectal artery embolization for treatment of symptomatic internal hemorrhoidal disease: an interactive systematic review of clinical trials
Source: CVIR Endovasc. 2024 May 11;7:45. doi: 10.1186/s42155-024-00458-2 (PMC11088570; doi:10.1186/s42155-024-00458-2)
Supplement: Supplementary file 1 — Supplementary Material 1. [file 42155_2024_458_MOESM1_ESM.docx]

**Supplement Content**

1. **Supplementary Figure 1.** Study Design Frequencies in Investigating Rectal Artery Embolization for Symptomatic Hemorrhoidal Disease.
2. **Supplementary Figure 2**. Type of embolic materials.
3. **Supplementary Figure 3.** Technical success rate.
4. **Supplementary Figure 4.** Procedural approach.
5. **Supplementary Figure 5**. Number of reinterventions needed.
6. **Supplementary Figure 6.** Follow up time points.
7. **Supplementary Figure 7.** Postoperative complications and clinical outcomes
8. **Supplementary Table 1.** Literature search details.
9. **Supplementary Table 2.** Overview of all included completed studies and description of common design, data elements, and core outcome measures reported.
10. **Supplementary Table 3. Procedural details and clinical outcomes.**
11. **Supplementary Table 4** Treatment Outcomes and Clinical Success Rates in Hemorrhoid Management.

**Supplementary Figure 1.** Study Design Frequencies in Investigating Rectal Artery Embolization for Symptomatic Hemorrhoidal Disease.

**Supplementary Figure 2**. Type of embolic materials

**Supplementary Figure 3.** Technical success rate.

**Supplementary Figure 4.** Procedural approach.

**Supplementary Figure 5**. Number of reinterventions needed.

**Supplementary Figure 6.** Follow up time points.

**Supplementary Figure 7**. Postoperative complications and clinical outcomes

**Supplementary Table 1.** Literature search details.

| **Search** | **Database** | **Query** | **Date** | **Results** | **Duplicate** | **Excluded** | **Included** |
| --- | --- | --- | --- | --- | --- | --- | --- |
| **1** | PubMed |  | Mar 25, 2023 | 40 | 0 | 29 | 11 |
| **2** | PubMed |  | Mar 25, 2023 | 57 | 5 | 47 | 5 |
| **3** | PubMed |  | Mar 25, 2023 | 468 | 91 | 377 | 0 |
| **4** | ClinicalTrials.gov | (Emborrhoid) OR (Embolization) AND (rectal) OR (haemorrhoidal) | Mar 25, 2023 | 74 | 4 | 64 | 1 |
| **5** | PubMed | (EMborrhoid) | Mar 25, 2023 | 24 | 21 | 2 | 0 |
| **6** | Europe PubMed Central | (Emborrhoid) OR (rectal artery) OR (hemorrhoidal artery) AND (embolization) OR (embolisation) | Mar 25, 2023 | 964 | 90 | 813 | 1 |
| **7** | PubMed | Rectal artery AND Embolization OR Embolisation OR Emborrhoid AND hemorrhoid | Mar 25, 2023 | 537 | 104 | 433 | 0 |
| **8** | PubMed | ((Rectal artery) AND (Embolization)) AND (Hemorrhoids) | Mar 27, 2023 | 138 | 138 | 0 | 0 |
| **9** | PubMed | Emborrhoid | Mar 27, 2023 | 23 | 23 | 0 | 0 |
| **10** | ClinicalTrials.gov | Emborrhoid | Mar 27, 2023 | 3 | 3 | 0 | 0 |
| **11** | Europe PubMed Central | ((Rectal artery) AND (Embolization)) AND (Hemorrhoids) | Mar 27, 2023 | 206 | 109 | 89 | 0 |
| **TOTAL** |  |  |  | 2534 | 588 | 1854 | 18 |

**Supplementary Table 2.** Overview of all included completed studies and description of common design, data elements, and core outcome measures reported.

| **Author** | **Zakharchenko A. et al. (2016)** | **Campenni P. et al. (2022)** | **Han X. et al. (2021)** | **Vidal V. et al. (2015)** | **Wang X. et al. (2021)** | **Iezzi et al. (2021)** | **Mousa N. et al. (2021)** | **De Gregorio, M.A et al. (2022)** | **Stecca T. et al. (2021)** |
| --- | --- | --- | --- | --- | --- | --- | --- | --- | --- |
| **Study design** | Cohort | Cohort | Cohort | Case series | Cohort | Pilot study | Cohort | Cohort | Cohort |
| **Control group** | None | None | None | None | None | None | None | None | None |
| **Number of participants** | 40 | 21 | 32 | 14 | 41 | 12 | 30 | 21 | 43 |
| **Gender (% female)** | 25 | 23.8 | 43.7 | 21.4 | 34.1 | 33.3 | 37 | 42.8 | 44.1 |
| **Status** | Completed | Completed | Completed | Completed | Completed | Completed | Completed | Completed | Completed |
| **Technical success** | N/A | Successful in all patients. | Successful in all patients. | Successful in 72% of patients. | Successful in all patients. | Successful in all patients. | Successful in 93% of patients. | Successful in all patients. | Successful in all patients. |
| **Type of embolization materials** | Metallic coils & Synthetic PVA particles | Interlock coils and Detachable Embolization Coils. | Gelatin Sponge Particles (350–560 μm) + Metallic Coils (2–3 mm) | Micro-Coils (0.018) | Metallic Coils (2–3 mm) + Gelfoam Particles (350–560 μm) | 0.018-inch Detachable Micro-Coils | Pushable Fiber Coils (2–3 mm) | Bare Platinum Detachable Micro-coil |  |
| **Approach** | Transfemoral | Trans radial | Transfemoral | N/A | Transfemoral | Transradial | Transfemoral | Transradial | Transfemoral |
| **Reintervention (number of patients)** | None | None | 4 | 4 | None | None | 4 | 4 | None |
| **Timepoints** | 1 month | 6, 12 months (Short-term follow-up) | N/A | 1 month (Clinical success) | a short-term follow-up period ranging from 6 to 15 months. | 6 hours after the procedure and 4-week follow-up (Discharge criteria) | Median follow-up of 5 months (5 months) | 3, 6, 12 months (Follow-up) | 7 days, 1 month, 6 months of (Follow-up) |
| **Adverse events (number of patients)** | N/A | 3 | 4 | 1 | 0 | 2 | 0 | 3 | 2 |

**Supplementary Table 2 (Continued).** Overview of all included completed studies and description of common design, data elements, and core outcome measures reported.

| **Author** | **Sun X. et al. (2018)** | **Puchol MD. et al. (2020)** | **Küçükay MB. et al. (2021)** | **Falsarella. et al. (2023)** | **Vidal V. (Assistance Publique Hopitaux De Marseille) (2018)** | **Tradi F. et al. (2018)** |
| --- | --- | --- | --- | --- | --- | --- |
| **Study design** | Cohort | Cohort | Cohort | Prospective randomized clinical trial | Cohort | Cohort |
| **Control group** | None | None | None | Ferguson closed hemorrhoidectomy surgical group | None | None |
| **Number of participants** | 23 | 20 | 42 | 33 | 14 | 25 |
| **Gender (% female)** | 60.87 | 20 | 28.5 | 40 | 21 | 36 |
| **Status** | Completed | Completed | Completed | Completed | Completed (not published) | Completed |
| **Technical success** | Successful in all patients. | Successful in 90% of patients. | 90-100% | Successful in all patients. | Successful in all patients. | Successful in 96% of patients. |
| **Type of embolization** | 3-5 mm metallic coils (Gianturco coils; Cook Europe, Bjaeverskov, Denmark) | PVA particles (300-500 µm) + coils (2-3 mm) | Microspherical tris-acryl gelatin microspheres (Embosphere; Merit Medical), 500–700, 700–900, and 900–1,200 μm in size. | 0.018-inch fibered coils (Interlock IDC; Boston Scientific Marlborough, Massachusetts) | pushable microcoils (0.018) | 2- or 3-mm-diameter microcoils (Nester; Cook Medical, Bloomington, Indiana). |
| **Approach** | Transfemoral | Transfemoral and Transradial | N/A | Transfemoral | Transfemoral | Transfemoral |
| **Reintervention (number of patients)** | None | 3 | 0 | 2 | 2 | 11 |
| **Timepoints** | 1 and 6 months. | Range from 2-18 months | 1 month, 3 months, 6 months, and 12 months | 1 month, 3 months, 6 months, and 12 months | N/A | 1-month, 3-month, and 6-month |
| **Adverse events (number of patients)** | 12 | 0 | 23 | 2 | N/A | 0 |

RBL: Rubber band ligatures. SRAE: Superior Rectal Artery Embolization. DG-HAL: Doppler-Guided Hemorrhoidal Artery Ligation.

**Supplementary Table 3. Procedural details and clinical outcomes.**

| **Study** | **Technical Success** | **Operating Time** | **Complications and Adverse Events** | **Clinical Success** | **Rebleeding** | **Additional Treatments** | **Postoperative Pain** | **Tenesmus** | **Patient Satisfaction** |
| --- | --- | --- | --- | --- | --- | --- | --- | --- | --- |
| **Wang** | 100% | (49 ± 10) min | None | 87.0% (Group A) vs. 88.9% (Group B) | 16.7% | PPH, rubber band ligation, inferior rectal artery embolization, sclerotherapy injection | 34.1% | 48.8%, resolving in 3-7 days | N/A |
| **Moussa** | N/A | N/A | None | 72% | N/A | N/A | N/A | N/A | N/A |
| **Tradi** | 100% | N/A | No major complications | 72% at 12 months | 28% | N/A | N/A | N/A | 86.7% at 12 months |
| **Falsarella** | 100% | N/A | Edema, acute fissure (Grade 2), surgical wound dehiscence (Grade 1), stenosis, skin tag, fissure | N/A | N/A | N/A | Pain during first bowel movement higher than embolization, medication use significantly lower | N/A | 86.7% at 30 days, 86.7% at 3 months, 71.5% at 6 months, 61.6% at 12 months |
| **Vidal** | 100% | N/A | No pain or ischemic complications, rebleeding in 2 patients | N/A | N/A | Additional embolization for rebleeding, one patient with painful perianal reaction | N/A | N/A | N/A |
| **X SUN** | 100% | 45 ± 8 min | None | 91.3% | 8.7% | Repeat embolization for rebleeding | N/A | Self-limited in 34.78% cases | N/A |
| **Lezzi** | N/A | 39.42 min | No significant intra/post-procedural discomfort | N/A | N/A | N/A | N/A | N/A | N/A |
| **De Gregorio** | 100% | N/A | No major complications, radial hematoma, minor tenesmus | Healing (85.7%), Improvement (9.5%), No changes (4.7%) | N/A | N/A | N/A | N/A | N/A |
| **Zakharchenko** | 100% | N/A | No observed hematomas, infections, or pseudoaneurysms | N/A | N/A | N/A | N/A | N/A | 83% with grade III hemorrhoids, 94% with grades I–II hemorrhoids |
| **Puchola** | 90% | N/A | Discharge: 24 hours for femoral approach, 6 hours for radial puncture. Technical failure in 2 cases: dissection of the inferior mesenteric artery and an infrarenal aortic aneurysm. Mean radiation dose: 535,060 mGy/cm2. Range: 66,532-1,445,794 mGy/cm2. Higher dose for radial access (mean 743,370.33 mGy/cm2) compared to femoral access (mean 514,244.60 mGy/cm2). | Clinical success achieved in 15 out of 18 patients (83.4%).. Mean follow-up: 10.6 months (range: 2-18 months). Complications and  No immediate major complications. 3 patients experienced clinical failure, requiring repeat procedure or surgery. | Recurrence of bleeding in 1 patient, requiring further embolization  2 patients had acute bleeding one month later, requiring surgical intervention. | N/A | Interventions: Two patients developed rectal pain post-treatment, relieved with paracetamol. Two patients reported rectal heaviness but did not require treatment. | N/A | N/A |
| **X. Han** | 100% | 45 ± 8 min | None | N/A | N/A | Repeat embolization for recurrence | N/A | 53.1% postoperative tenesmus | N/A |
| **Stecca** | 100% | N/A | Hemorrhoidal crisis, hematoma at the puncture site (Clavien-Dindo Grade 1) | 92% Grade 2 at 1-month, 84% Grade 2 at 6-month | N/A | N/A | N/A | N/A | N/A |
| **Küçükay** | 100% | N/A | Small ulcerations, rectosigmoid junction ulcerations, fibrotic scar tissue | 93% at 12 months | N/A | N/A | N/A | N/A | N/A |
| **Campennì** | 100% | 40-50 min | Ecchymosis, arm pain, pseudoaneurysm (14.3% overall complication rate) | Decreased post-defecation bleeding, mean hemoglobin increase, 14.3% recurrence | N/A | N/A | N/A | N/A | N/A |

| **Author** | **Sun X. et al. (2018)** | **Puchol MD. et al. (2020)** | **Küçükay MB. et al. (2021)** | **Falsarella. et al. (2023)** | **Vidal V. (Assistance Publique Hopitaux De Marseille) (2018)** | **Tradi F. et al. (2018)** |
| --- | --- | --- | --- | --- | --- | --- |
| **Clinical Success and outcome assessment** | **Clinical success was observed in 21/23 (91.3%) patients.**  **GPS:-**  Grade II 6 (26.09%)  Grade III 17 (73.91%) | **Clinical success (number of patients and percentage)**  **15/18 (83.4%)**  **Rectal heaviness**  **2/18**  **Pain 3/10 VAS: -**  **2/18** | **Clinical success was achieved in 39 of 42 patients (93%)**  **at 12 months.**  **FBS: -**  significant decrease in the FBS at  12 months  **VAS: -**  significant decrease in the VAS score at 12  months  **QOLS: -**  significantly improved in all groups at 12  months  **GPS: -**  The GPS did not significantly change between the time points and between the groups (P > .05). | **Treatment satisfaction**  **In the embolization group, satisfaction (very satisfied or satisfied) with the treatment was: -**   - **86.7% (P = .218) at 30 days,** - **86.7% (P = .828) at 3 months,** - **71.5% (P =.253) at 6 months, and** - **61.6% (P = .023) at 12 months.**   **VAS: -**  the distribution of pain on Days 1, 3, and 7 revealed that the pain observed in the embolization group was statistically lower than that observed in the surgery group | **NA** | **Clinical success was obtained in 18 patients (72%),**  **GPS: -**  Stage II 17 (68%)  Stage III8 (32%)  **VAS: -**  score decreased from 4.6 to 2.3 (P < .01  **Bleeding score: -**  decreased from 5.5 to 2.3 (P < .01).  **QOLS and prolapse scores: -**  showed improvement (P <.05), |

**Supplementary Table 4** Treatment Outcomes and Clinical Success Rates in Hemorrhoid Management

**Supplementary Table 4 (Continued)** Treatment Outcomes and Clinical Success Rates in Hemorrhoid Management

| **Author** | **Zakharchenko A. et al. (2016)** | **Campenni P. et al. (2022)** | **Han X. et al. (2021)** | **Vidal V. et al. (2015)** | **Wang X. et al. (2021)** | **Iezzi et al. (2021)** | **Mousa N. et al. (2021)** | **De Gregorio, M.A et al. (2022)** | **Stecca T. et al. (2021)** |
| --- | --- | --- | --- | --- | --- | --- | --- | --- | --- |
| **Clinical Success and outcome assessment** | **NA** | **(HDSS) and (SHS)**  **were completed before the procedure.**  At the 12-month follow-up visit,  **HDSS: -**  decreased from 11.1 ± 4.2 to 4.7 ± 4.6 (p < 0.0001)  **SHS: -**  decreased from 18.8 ± 4.8 to 10.2 ± 4.9 (p < 0.0001), with a significant improvement of hemorrhoidal symptoms, except for itching.  **GPS:-**  Grade II 8 (38.1%)  Grade III 10 (47.6%)  Grade IV 3 (14.3%) | **Bleeding symptoms resolved in 27 (84.4%) patients.**  **GPS: -**  Stage II 12 (37%)  Stage III 20 (63%) | **NA** | **The preliminary clinical efficacy (87.0% vs 88.9%) showed no significant difference between the 2 groups (p = 0.098).**  **FBS: -**  The post-operative FBS was significantly lower than the pre-operative bleeding severity score in each group (p < 0.01, respectively)  **VAS: -**  were between 1 and 6 (mean 2.02 ± 2.20) on the day of operation,  between 0 and 3 (mean 0.37 ± 0.70) on the second day after surgery.  **GPS: -**  grade II internal hemorrhoidal 16 (39%),  grade III 25 (61%) | **The mean Rorvik score =(HDSS) and (SHS)**  At the follow-up visit, the mean Rorvik score (HDSS & SHS-HD) decreased from 31.50 (7.50) to 13.11 (8.33) (p<.001). | **Clinical score improvement was observed in 72%.**  **No improvement in bleeding was observed in eight patients (28%).**  **FBS: -**  In 72% of patients, we observed clinical success with an improvement by at least 2 points.  **General symptom score**  **QOLS**  **GPS: -**  was not modified by embolization. Of note, the frequency of severe prolapse was already low in our patient population before treatment | **Clinical success rates range from 63% to 97%**  **GPS: -**   - Grade I 3 (14.2%) - Grade II 15 (71.4%) - Grade III3 (14.2%) - Grade IV 0     **FBS: -**  Change (95% CI) 4.2 (5.4–1.1)  **VAS: -**  Change (95% CI) 4.2 (4.9–1.3)  **QOLS: -**  Change (95% CI) 1.7 (2.2–0.7)  p Value 0.0001 | **FBS: -**  significantly reduced (P <0.001).  **The bleeding score: -**  significantly reduced (P ¼.001).  **QOLS: -**  significantly improved (P ¼.001). |

***French Bleeding Score (FBS)**

*** Visual analog scale (VAS)**

*** Quality of life scale (QOLS)**

*** Goligher Score (GPS)**

*** The Hemorrhoidal Disease Symptom Score (HDSS)**

***Short Health Scale (SHS)**
